# An unusual case of *Streptococcus pyogenes* infective endocarditis demonstrating the usefulness of repeated echocardiography

**DOI:** 10.1016/j.idcr.2026.e02539

**Published:** 2026-03-05

**Authors:** Gabriel Hirdman, Anders Roijer, Magnus Paulsson, Per Wierup, Magnus Rasmussen

**Affiliations:** a Department of Clinical Sciences, Lund University, Lund, Sweden; b Department of Cardiology, Clinical Sciences, Lund University and Skåne University Hospital, Lund SE-221 85, Sweden; c Department of Clinical Microbiology, Skåne University Hospital, Lund, Sweden; d Division of Infection Medicine, Department of Clinical Sciences Lund, Lund University, Lund, Sweden; e Department of Cardiothoracic and Vascular Surgery, Skåne University Hospital, Lund, Sweden; f Department of Infectious Diseases, Skåne University Hospital, Lund, Sweden

**Keywords:** Infective endocarditis, *Streptococcus pyogenes*, Embolization, Heart valve surgery

## Abstract

Infective endocarditis (IE) is most often caused by alpha-hemolytic streptococci or *Staphylococcus aureus* and is characterized by the presence of vegetations on the heart valves. Here we present a case of IE caused by *Streptococcus pyogenes,* a distinctly uncommon IE-pathogen, where no vegetations could be visualized on repeated echocardiography. Diagnosis was instead evident from septic embolizations and progressive aortic insufficiency and IE was verified upon heart valve surgery. The patient was a 69-year-old man who presented with a two-day-history of fever and confusion. Several lesions on the skin and in the brain were suggestive of septic embolization and blood cultures grew *S. pyogenes* of sequence type 39, *emm4.* The patient developed progressive aortic insufficiency and was subjected to surgery at day seven after admittance. The aortic cusp showed signs of destructive IE and analysis of the valve demonstrated the presence of DNA from *S. pyogenes* ultimately confirming the diagnosis. The patient received a biological heart valve prosthesis and needed medical treatment for atrial fibrillation and heart failure post operatively. There was no relapse at six months post-surgery.

## Introduction

Infective endocarditis (IE) is a severe infection of the heart valves typically featuring a vegetation, composed of bacteria and host cells, at the surface of the affected valve [1]. Parts of this vegetation can embolize and cause damage to distant organs. The vegetation can be visualized by echocardiography which together with the presence of typical IE-bacteria in blood cultures constitute the two major Duke criteria for IE diagnosis [2]. The infection can also damage the heart valve leading to regurgitation and heart failure [1].

The most common bacterium causing IE is *Staphylococcus aureus* which typically cause IE with acute onset and a severe course of infection [3]. Viridans streptococci are also common IE pathogens and have a subacute onset of infection and a better prognosis [4]. Beta-hemolytic streptococci rarely cause IE but when they do, the onset of symptoms is typically acute and the course is often severe [4], [5], [6]. Of the beta-hemolytic streptococci, both *Streptococcus agalactiae* and *Streptococcus dysgalactiae* are rare causes of IE [5], [7], [8], [9], but are still regarded as typical IE pathogens [2]. However, *Streptococcus pyogenes* is a very rare cause of IE and not considered typical for IE [5], [7], [8], [9], [10], [11]. This means that three positive blood cultures are needed for *S. pyogenes* bacteremia to be counted as major criterion for IE [2].

Oppegaard and coworkers described seven cases of IE caused by *S. pyogenes* and noted that the isolates were of uncommon *emm-*types [11]. Interestingly *S. pyogenes* isolates from IE adhered better to fibronectin and carried more genes encoding fibronectin-binding surface proteins than non-IE isolates suggesting that fibronectin-binding might be an important factor for *S. pyogenes* causing IE.

In this report we describe a case of *S. pyogenes* IE where the diagnosis became evident through embolizations and progressive aortic valve regurgitation despite that no vegetations could be visualized by transesophageal echocardiography

## Case presentation

A 69-year-old man presented to the emergency department with a three-day history of fever and altered mental status, along with new-onset left-sided weakness and facial drooping. Although the patient´s wife reported that he had been mostly bedridden, he stated that he felt well and denied all symptoms described. His previous medical history was unremarkable, and he was not taking any medications. There was no history of substance misuse, and he had no prior encounters with the hospital system.

**Fig. 1:**
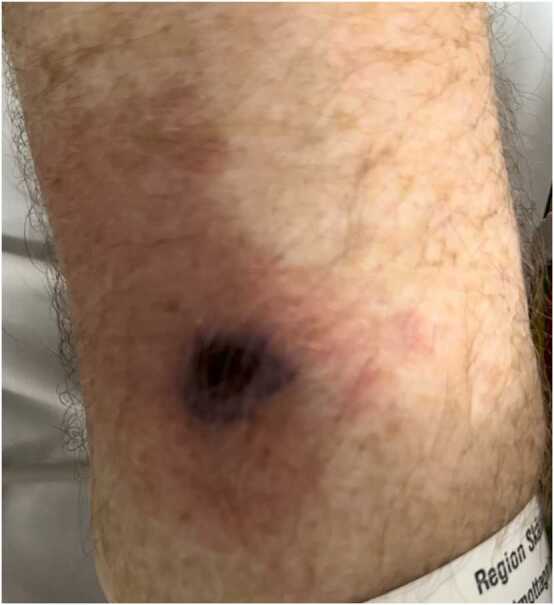
Picture of the partly necrotic inflamed area suspected as septic embolization at the left forearm.

**Fig. 2:**
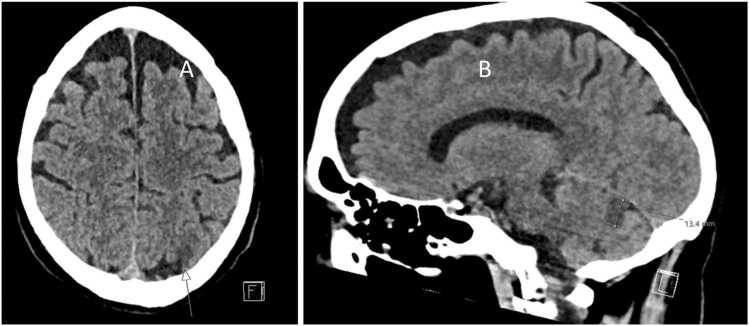
Computerized tomography of the brain demonstrating signs of ischemic injuries. In A is a horizontal view where the arrow indicates the site of injury. In B is a sagittal view where the injury to the cerebellum is marked.

On examination, he was oriented to person but not to time or place. On auscultation, his lungs were clear, and cardiac auscultation revealed an irregular rhythm without murmurs. Inspection of the skin revealed suspected septic emboli on the first digit of the right foot and the left forearm (Fig. 1). No nuchal stiffness was noted. He had a respiratory rate of 33 breaths per minute, oxygen saturation of 94% on room air, an irregular pulse ranging from 75 to 170 beats per minute, blood pressure of 122/70 mmHg, and his body temperature was 38.5°C. An electrocardiogram (ECG) revealed new-onset atrial fibrillation. Laboratory studies revealed significant systemic inflammation with leukocytosis (22 × 10⁹/L, normal range: 3.5–8.8 × 10⁹/L) and an elevated C-reactive protein (CRP) of 227 mg/L (normal: <5 mg/L). Serum creatinine was elevated at 149 μmol/L (normal range: 60–105 μmol/L), while hemoglobin was within normal limits at 136 g/L (normal range: 134–170 g/L). Other abnormalities included an elevated high-sensitivity troponin I of 2745 ng/L (normal: <54 ng/L), NT-proBNP of 8612 ng/L (normal: <125 ng/L), and lactic acid of 3.8 mmol/L (normal: 0.5–2.2 mmol/L). Urine dipstick testing revealed proteinuria and hematuria. Blood, urine, and nasopharyngeal swab cultures were obtained. The patient was empirically instituted on intravenous meropenem (2 g), aciclovir (500 mg), and betamethasone (8 mg) for coverage of meningitis, encephalitis, and for IE. Intravenous fluids were started for suspected prerenal acute kidney injury. An urgent computerized tomography of the brain revealed two subacute ischemic lesions, one measuring 10 mm in the posterior part of the left parieto-occipital lobe and one of 10 mm anteromedially in the left cerebellum (Fig. 2).

Upon admission to the ward, a transthoracic echocardiography (TTE) was performed. The left ventricle was mildly hypertrophic with mild dilation and a reduced ejection fraction. There was mild aortic regurgitation and no vegetations were observed. Preliminary blood culture results revealed gram-positive cocci in chains. Given the clinical picture and imaging findings, suspicion of meningitis and encephalitis was ruled out, and antibiotic treatment was switched to intravenous ampicillin (3 g every 6 h) for suspected IE.

**Fig. 3:**
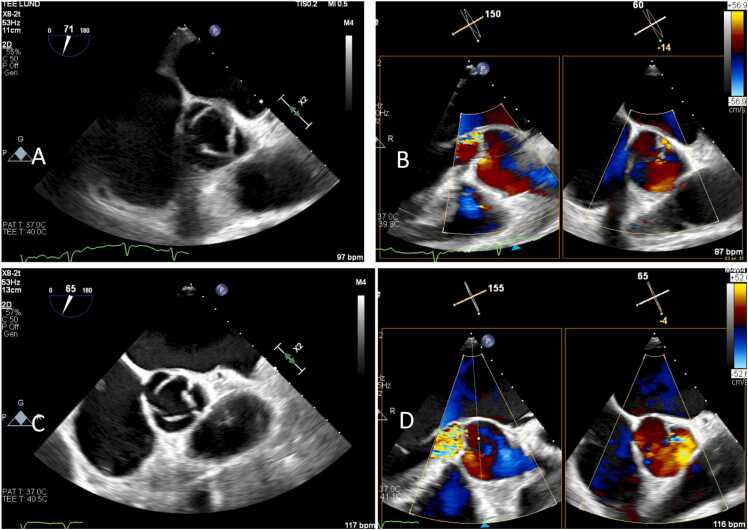
In A, TEE demonstrating the normal aortic valve is shown. In B, TEE demonstrating a mild aortic regurgitation from the commissure non-coronar and left aortic cusp of the aortic valve is shown. In C, TEE from day 7 of hospitalization showing partial destruction of the left aortic cusp. In D, TEE from day 7 of hospitalization demonstrating severe aortic regurgitation with origin from the left aortic cusp.

The following day, a transesophageal echocardiography (TEE) was performed. An eccentric jet over the aortic valve was detected, indicating mild aortic insufficiency. No vegetations were observed but the left coronary cusp was slightly irregular at the free edge compared to the other cusps (Fig. 3a and b). Additionally, a patent foramen ovale was identified. Shortly afterward, all four blood culture flasks returned positive for *S. pyogenes.* Nasal swab also grew *S. pyogenes*, and further history-taking revealed that the patient had experienced throat pains during the week prior to admission. Antibiotic treatment was changed to IV benzylpenicillin (3 g every 6 h). A computerized tomography of the chest was performed, demonstrating no additional foci of infection.

The multidisciplinary IE team at Skåne University hospital was consulted to review the patient. Although the clinical presentation raised suspicion for IE with suspected septic embolizations to the skin and brain, diagnostic criteria for IE were not fulfilled. Importantly, there were no vegetations of the heart valves, and *S. pyogenes* is not a typical IE pathogen according to the Duke-ISCVID diagnostic criteria [2]. A follow-up TEE was scheduled in four days.

The patient showed gradual improvement in both laboratory parameters and clinical condition, including neurological status. Leukocytosis resolved and CRP showed a decline from 227 mg/L to 54 mg/L. Renal function improved with creatinine decreasing to 73 μmol/L. On day 6 after admission, the scheduled follow-up TEE was performed, revealing partial destruction of the left aortic cusp leading to severe aortic insufficiency (Fig. 3c and d). Furthermore, wound fluid obtained from a punch biopsy of a suspected new septic embolus on the left thigh grew *S. pyogenes*.

**Fig. 4:**
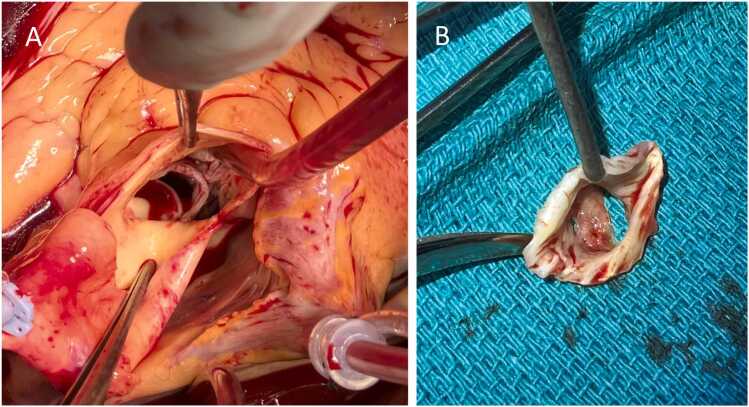
A is an intraoperative picture showing the aortic root exposed through a standard aortotomy. The patient has a functional bicuspid aortic valve. The aortic annulus is intact with no signs of abscess. In B is a picture of the aortic valve, excised *en bloc.* The large vegetation is well visualized.

Given these findings, the multidisciplinary IE team reconvened and concluded that the diagnostic criteria for IE were now met, and a decision was made to proceed with surgical intervention due to the uncontrolled infection in combination with severe aortic insufficiency. Preoperative coronary angiography was without significant stenoses. Surgery was conducted on day 7 after admission. Intraoperatively, the left aortic cusp was found to have a large perforation, characteristic of IE (Fig. 4). An Edwards Inspiris Resilia biological valve (Edwards Lifesciences, Irvine, California, U.S), was implanted, and an AtriClip (AtriCure, Mason, Ohio, U.S) was placed to eliminate the need for long-term anticoagulation. Additionally, the foramen ovale was closed. The postoperative period in the intensive care unit was uneventful. A postoperative TTE confirmed normal left ventricular function and a well-functioning prosthetic valve. 16S rRNA gene PCR and sequencing of material from the excised valve was positive for *S. pyogenes* DNA; however, cultures remained negative.

The isolate from blood was subjected to whole genome sequencing using the standard protocols of our laboratory revealing that the isolate was of sequence type 39. The gene encoding the M protein was of the *emm4.0* type. Several genes encoding fibronectin binding proteins (prtf1, gfba, sof and fbaA) were identified in the genome.

The postoperative period was uncomplicated. Throughout the 28-day intravenous benzylpenicillin course, inflammatory markers steadily decreased. By day 26, CRP had declined to 37 mg/L, and leukocytes remained stable at 4.2 × 10⁹/L. During follow-up visits he reported continued clinical improvement. He remained afebrile, and a repeat chest radiograph showed further regression of the pleural effusion. His recovery trajectory remained positive, with no signs of infection relapses, although he experienced atrial fibrillation relapse. The medical treatment for heart failure was optimized at an outpatient cardiology department.

## Discussion

This case is unusual both in that the causative bacterium is uncommon and in that no vegetations could be visualized at echocardiography. The initial presentation of the patient with stroke, fever and skin lesions led us to suspect IE but the picture was blurred by the finding of *S. pyogenes* in blood and the lack of vegetations on the valve at echocardiography. Importantly, however, we repeated echocardiography and could thus monitor the rapid destruction of valve tissues which necessitated surgical exchange of the aortic valve.

*S. pyogenes* is an important human pathogen that can cause a variety of localized infections [12]. The patient disclosed that he had had severe throat pains in the week preceding the hospitalization and we hypothesize that this tentative pharyngitis was caused by the same bacterium. IE, however, is an exceedingly rare presentation of invasive *S. pyogenes* infection. Previous work has indicated that isolates of *S. pyogenes* belonging to certain *emm-*types with the ability to bind to immobilized fibronectin have an increased likelihood of causing IE [11]. The isolate causing the present episode was of the *emm4* type which was the cause of 7% of invasive *S. pyogenes* infections in our area [13]. *Emm4* isolates belong to serum opacity factor positive types of *S. pyogenes* which typically bind to fibronectin [14]. The gene encoding the fibronectin-binding serum opacity factor [15] was identified in the genome as well as genes encoding other surface-associated fibronectin-binding protein such as protein F [16], FbaA [17] and GfbA [18]. Our findings align with those of Oppegaard and coworkers suggesting that fibronectin-binding may contribute to the pathogenesis of *S. pyogenes* IE.

We were initially puzzled by the combination of signs of embolization to the brain and skin in the absence of valve vegetations. However, *S. pyogenes* is known to interact with platelets possibly causing relatively large emboli despite that the mass of bacteria on the heart valves was not visible at echocardiography [19]. Moreover, the aortic valve became incompetent during the course of the disease indicating that the bacterium had tissue destructing capabilities. *S. pyogenes* is known to secrete and activate a number of proteolytic enzymes that can have participated in this process [20].

In conclusion, our case demonstrates that repeated echocardiography might be useful in cases of suspected IE where initial evaluation does not confirm the diagnosis and that *S. pyogenes* can cause destructive IE.

## Author statement

The authors certify that they have obtained the consent of the patient to publish this report. The patient understands that his identity will not be revealed through the figures presented.

## CRediT authorship contribution statement

**Per Wierup:** Writing – review & editing, Formal analysis. **Magnus Rasmussen:** Writing – review & editing, Writing – original draft, Supervision, Resources, Project administration, Methodology, Investigation, Formal analysis, Data curation, Conceptualization. **Anders Roijer:** Writing – review & editing, Formal analysis. **Magnus Paulsson:** Writing – review & editing, Formal analysis. **Hirdaman Gabriel:** Writing – review & editing, Writing – original draft, Investigation.

## Funding

There was no specific funding for this study.

## Declaration of Competing Interest

The authors declare that they have no known competing financial interests or personal relationships that could have appeared to influence the work reported in this paper.
